# Interface Properties between Lithium Metal and a Composite Polymer Electrolyte of PEO_18_Li(CF_3_SO_2_)_2_N-Tetraethylene Glycol Dimethyl Ether 

**DOI:** 10.3390/membranes3040298

**Published:** 2013-10-25

**Authors:** Hui Wang, Masaki Matsui, Yasuo Takeda, Osamu Yamamoto, Dongmin Im, Dongjoon Lee, Nobuyuki Imanishi

**Affiliations:** 1 Graduate School of Engineering, Mie University, Tsu, Mie 514-8507, Japan; E-Mails: 411db02@m.mie-u.ac.jp (H.W.); matsui@chem.mie-u.ac.jp (M.M.); takeda@chem.mie-u.ac.jp (T.T.); imanishi@chem.mie-u.ac.jp (N.I.); 2 Samsung Advanced Institute of Technology, Samsung Electronics, Yongin 446-712, Korea; E-Mails: dongmin.im@samsung.com (D.I.); dongjoon.lee@samsung.com (D.L.)

**Keywords:** composite solid polymer electrolyte, tetraethylene glycol dimethyl ether, water-stable lithium electrode, lithium dendrite, aqueous lithium-air battery

## Abstract

The electrochemical properties of a composite solid polymer electrolyte, consisting of poly(ethylene oxide) (PEO)-lithium bis(trifluoromethanesulfonyl)imide (LiTFSI) and tetraethylene glycol dimethyl ether (TEGDME) was examined as a protective layer between lithium metal and a water-stable lithium ion-conducting glass ceramic of Li_1__+*x*+*y*_(Ti,Ge)_2−*x*_Al*_x_*P_3−*y*_Si*_y_*O_12_ (LTAP). The lithium ion conductivity and salt diffusion coefficient of PEO_18_LiTFSI were dramatically enhanced by the addition of TEGDME. The water-stable lithium electrode with PEO_18_LiTFSI-2TEGDME, as the protective layer, exhibited a low and stable electrode resistance of 85 Ω·cm^2^ at 60 °C, after 28 days, and low overpotentials of 0.3 V for lithium plating and 0.4 V for lithium stripping at 4.0 mA·cm^−2^ and 60 °C. A Li/PEO_18_LiTFSI-2TEGDME/LTAP/saturated LiCl aqueous solution/Pt, air cell showed excellent cyclability up to 100 cycles at 2.0 mAh·cm^−2^.

## 1. Introduction

Aqueous lithium-air batteries have been considered as a promising electrochemical energy storage system for electric vehicles. The theoretical energy density for the cell reaction of 4Li + 6H_2_O + O_2_ = 4(LiOH·H_2_O) is 1910 Wh·kg^−1^, which is significantly higher than 387 Wh·kg^−1^ for typical lithium-ion batteries with a carbon anode and LiCoO_2_ cathode. The aqueous lithium-air battery system, proposed by Imanishi and co-workers, is composed of a lithium metal electrode, a lithium ion conducting polymer electrolyte based on poly(ethylene oxide) (PEO)*_x_*-lithium bis(trifluoromethanesulfonyl)imide (LiTFSI), a water-stable lithium ion-conducting glass ceramics of Li_1__+*x*+*y*_(Ti,Ge)_1__−*x*_Al*_x_*P_3−*y*_Si*_y_*O_12_ (LTAP), an aqueous electrolyte with saturated LiCl, and a carbon air electrode [1]. At present, the only acceptable water-stable lithium ion conducting solid electrolyte is LTAP, which exhibits a lithium ion conductivity of higher than 10^−4^ S·cm^−1^ at ambient temperature, and is stable in saturated LiOH and LiCl aqueous solution, but exhibits poor chemical stability towards lithium metal [2]. To construct a water-stable lithium electrode (WSLE) based on LTAP requires a chemically stable lithium conducting electrolyte interlayer between the lithium metal and LTAP. Some candidates for this interlayer have been adopted, such as lithium nitride [3], lithium phosphorous oxynitride [4], and polymer electrolytes [5]. The two former materials are generally prepared by vapor deposition, which involves considerable cost and makes the preparation of large sized cells difficult. In contrast, the polymer electrolyte is stable in contact with lithium metal and large sized sheets can be easily fabricated. 

In our previous studies [6,7,8], we have reported that the interface resistance between lithium metal and polymer electrolytes in a WSLE is the dominant part of the cell resistance and an important factor in initiating lithium dendrite formation. The addition of ionic liquids into PEO_18_LiTFSI reduced the interface resistance and suppressed lithium dendrite formation [7]; however, high overpotentials for lithium deposition and stripping reaction were observed at high current densities. The WSLE should be operated at the highest current density possible with low overpotentials to prevent the formation of lithium dendrites [9]. The addition of low-molecular weight plasticizers to the PEO-based electrolyte is expected to improve the interface properties between lithium metal and the polymer electrolyte, and enhance the lithium ion transport number of the polymer electrolyte. Kim *et al*. [10] reported the use of low molecular weight oligomer ethers as a plasticizer to enhance the transport properties of polymer electrolytes, and Naoi *et al*. reported that the addition of poly(ethylene glycol) dimethyl ether (PEGDME) into propylene carbonate (PC) with LiClO_4_ suppressed dendrite formation [11]. Moreover, Yoshida *et al*. reported that the oxidative stability of glyme molecules is enhanced by complex formation with LiTFSI in a molar ratio of 1:1 [12]. Recently, Jung *et al*. [13] demonstrated a Li/tetraethylene glycol dimethyl ether (TEGDME)-LiCF_3_SO_3_/carbon, O_2_ cell capable of operating over many cycles with capacity and rate values as high as 5000 mAh·g^−1^ and 0.5 A·g^−1^, respectively. We have previously reported an increase in the lithium ion transport number and decreases in the interface resistance of the lithium metal and polymer electrolyte by the addition of PEGDME to PEO_18_LiTFSI, where the mean molecular weight of PEGDME was 500 [14]. Naoi *et al*. [11] concluded that the Gibbs activation energy for the charge transfer reaction on the lithium metal surface in PC-PEGDME-LiClO_4_ decreased with a decrease in the molecular weight of PEGDME in the range of 90–400. In this study, the interface resistance between lithium and the polymer electrolyte, and the transport properties of the PEO_18_LiTFSI-*x*TEGDME (M = 222.28 g·mol^−1^) composite polymer electrolyte (CPE) are examined as a function of the amount of TEGDME (*x*). In addition, the electrochemical performance of a Li/PEO_18_LiTFSI-2TEGDME/LTAP/saturated LiCl aqueous solution/Pt, air cell is evaluated at 60 °C. 

## 2. Results and Discussion

### 2.1. Evaluation of CPEs

**Figure 1:**
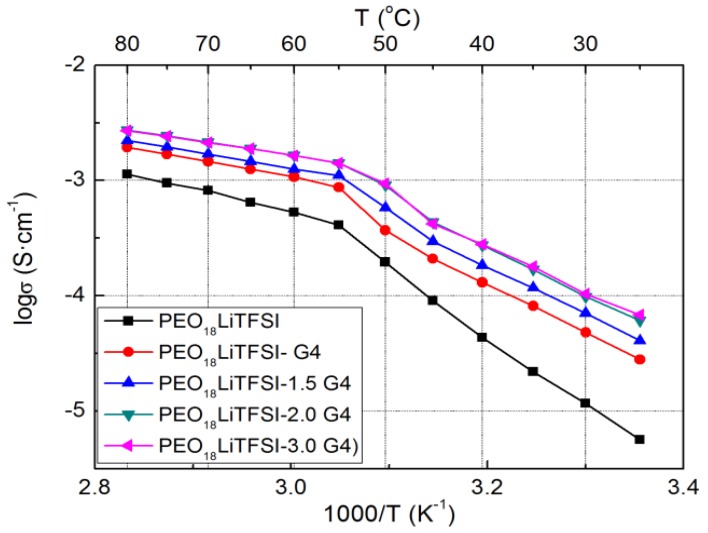
Temperature dependence of the electrical conductivity for PEO_18_LiTFSI-*x*G4 as a function of *x*.

The electrical conductivities of PEO_18_LiTFSI-*x*G4 (TEGDME abbreviated as G4 hereafter) were measured as a function of *x* (the molar ratio of G4 to ethylene oxide). Figure 1 presents Arrhenius plots for the conductivity of PEO_18_LiTFSI-*x*G4. The conductivity increases significantly with increasing *x* up to 2 and there is little difference in conductivity between the samples with *x* = 2 and 3. The electrical conductivity of PEO_18_LiTFSI-2G4 at 25 °C is almost one order of magnitude higher and that at 60 °C is three times higher than that of PEO_18_LiTFSI. TEGDME loosens the coordination of lithium ions with EO units in the PEO matrix and thus enhances the mobility of ions, and could also enable lithium ions to decouple from ion pairs, as shown by Kriz *et al*. [15]. The activation energies for conduction in PEO_18_LiTFSI-*x*G4 at high temperature decrease with increasing *x*. That of PEO_18_LiTFSI-2G4 was calculated to be 25.3 kJ·mol^−1^, in the range of 55 to 80 °C, which is lower than that for PEO_18_LiTFSI (38.8 kJ·mol^−1^) and that for PEO_18_LiTFSI-2.0 *N*-methyl-*N*-propylpiperdinium bis(fluorosulfonyl) imide (PP13FSI) (33.8 kJ·mol^−1^) [16].

**Figure 2:**
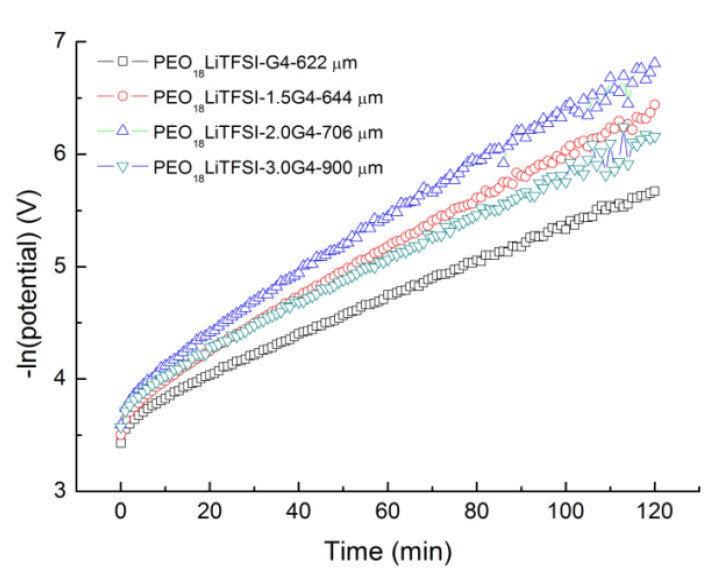
Natural logarithm of potential *vs*. time curves for the Li/PEO_18_LiTFSI-*x*G4/Li cells at 60 °C.

The overpotentials between lithium metal and polymer electrolytes for lithium deposition and stripping generally increase with current density and undergo a sudden increase with the polarization period at a current density (limiting current density). When the ionic concentration in the vicinity of cathode drops to zero, the limiting current density is reached and dendrite growth is initiated (Sand time) [17]. To operate the Li/PEO_18_LiTFSI/LTAP/Pt, air cell at high current density, it is necessary to enhance the limiting current density of lithium-ion conducting PEO-based polymer electrolytes (*I*_l_), which is proportional to the salt diffusion coefficient and lithium ion transference number, as shown by Equation (1):

*I*_l_ = 2*eC*_o_*D*/*t*_a_*L* (1)

where *C*_o_ is the initial concentration, *e* is the elemental charge, *D* is the salt diffusion coefficient, *t*_a_ is the anion transport number, and *L* is the thickness of the electrolyte. The salt diffusion coefficient of PEO_18_LiTFSI-*x*G4 was estimated using the method proposed by Ma *et al*. [18] as a function of *x*. Typical curves for the natural logarithm of potential *versus* time for the Li/PEO_18_LiTFSI-*x*G4/Li cells at 60 °C are shown in Figure 2, where the cells were polarized at 50 mV prior to the potential being interrupted. A distinct linear relation, which corresponds to the linear diffusion region as the concentration gradient of the cell relaxes, is observed for all cells after sufficient time. The salt diffusion coefficient (*D*) of PEO_18_LiTFSI-*x*G4 was calculated from the slope of the linear curves using Equation (2):

Slope = −π^2^*D*/*L*^2^ (2)

The calculated *D* are summarized in Table 1 along with the electrical conductivity results. A maximum salt diffusion coefficient of 3.37 × 10^−7^ cm^2^·s^−1^ was determined for *x* = 2.0, which is almost one order of magnitude higher than that for PEO_18_LiTFSI (3.60 × 10^−8^ cm^2^·s^−1^) and PEO_18_LiTFSI-1.44PP13FSI (2.25 × 10^−8^ cm^2^ s^−1^) [16], and four times higher than that for PEO_18_LiTFSI-18 wt % PEGDME (8.38 × 10^−8^ cm^−1^ s^−1^) [14]. 

**Figure 3:**
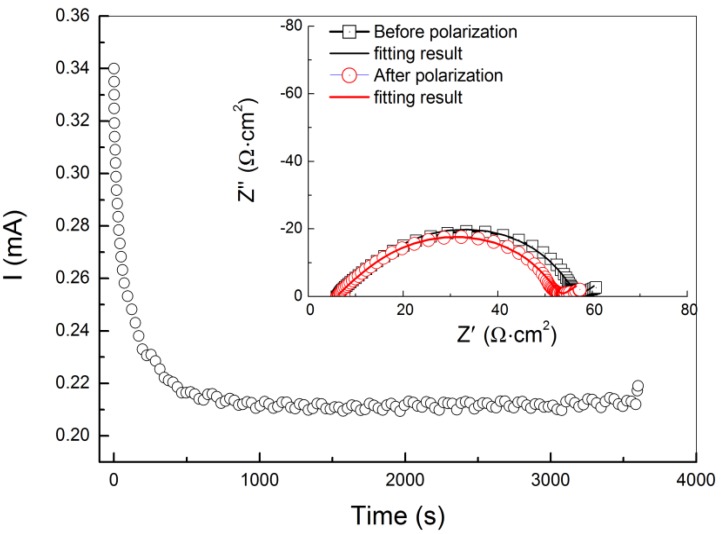
Steady-state method for lithium ion transference number determination. The symmetric Li/PEO_18_LiTFSI-2G4/Li cell current decays with time upon application of a DC bias of 20 mV until steady-state is reached. Corresponding impedance measurements performed at the initial state and steady-state, and the fitting results are shown in the inset.

Lithium ion transference numbers for PEO18LiTFSI-*x*G4 were measured using the method reported by Evans and Vincent [19]. Figure 3 shows a typical cell current decay curve upon application of a DC bias of 20 mV and impedance profiles for the Li/PEO18LiTFSI-2G4/Li cell. From these results, *t*_a_ was calculated using the Evans and Vincent equation, and the results are summarized in Table 1. Significant increase of the lithium ion transport number (*i.e.*, decrease of the anion transport number) was observed by addition of G4 into PEO_18_LiTFSI. The lithium ion transport number for PEO_18_LiTFSI-2G4 is slightly higher than that for LiTFSI-2G4. Based on these results for *D* and *t*_a_, the limiting current density (*I*_l_) for PEO_18_LiTFSI-*x*G4 was calculated and the results are summarized in Table 1, where the thickness (*L*) was 100 μm. PEO_18_LiTFSI-2G4 exhibits the highest *I*_l_ of 15.8 mA·cm^−2^, which is higher than that for PEO_18_LiTFSI by a factor of approximately 15. The high limiting current density of PEO_18_LiTFSI-2G4 suggests that a WSLE with PEO_18_LiTFSI-2G4 could be operated at a high current density.

**Table 1:** Ionic transport properties of PEO_18_LiTFSI-*x*TEGDME.

PEO_18_LiTFSI- *x*TEGDME	25 °C(×10^6^ S·cm^−1^)		60 °C(×10^4^ S·cm^−1^)		*E*a (kJ·mol^−1^)		*D* (×10^7^ cm^2^·s^−1^)	*t*_Li_^+^	*I*_l_ (mA cm^−2^) *L* = 100 μm
	σ	σ_Li_^+^	σ	σ_Li_^+^	Low temp. region	High temp. region			
*x* = 0	5.64	1.35	5.29	1.27	115.7	38.8	0.36	0.24	1.02
*x* = 1.0	28.0	12.0	10.7	4.60	99.8	30.7	1.05	0.43	3.97
*x* = 1.5	40.7	18.3	12.5	5.62	82.4	27.1	1.50	0.45	5.87
*x* = 2.0	60.8	32.8	16.5	8.91	78.2	25.3	3.37	0.54	15.8
*x* = 3.0	68.3	39.6	16.4	9.51	72.9	25.0	2.27	0.58	11.6

**Figure 4:**
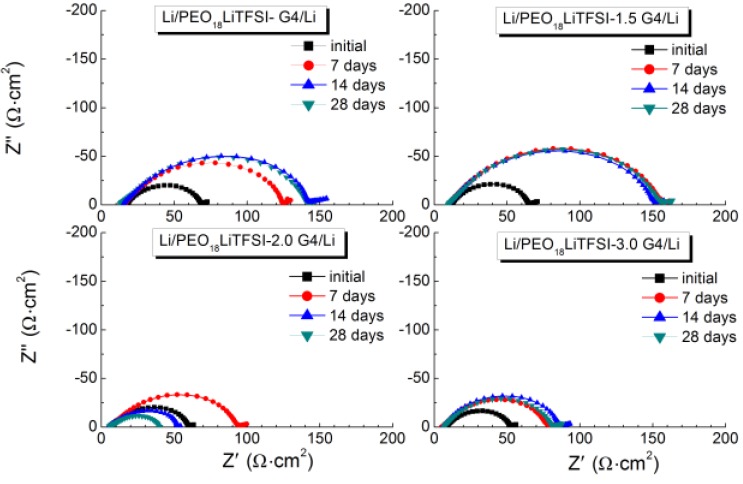
Impedance spectra of PEO_18_LiTFSI-*x*G4 as a function of the storage time at 60 °C.

**Figure 5:**
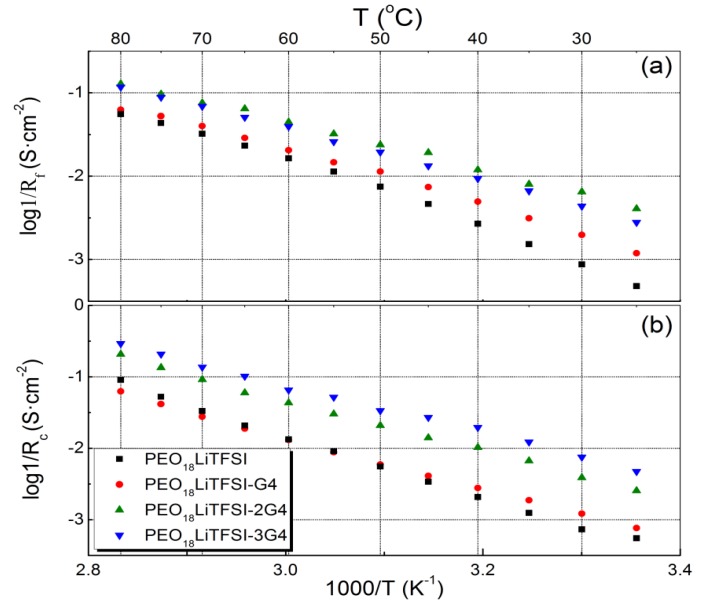
Temperature dependence of the inverse of (**a**) the passivation film resistance; and (**b**) the charge transfer resistance for Li/PEO_18_LiTFSI-*x*G4/Li.

Low and stable interface resistance between lithium metal and polymer electrolytes is essential for the electrochemical performance of WSLEs and to reduce lithium dendrite formation [7]. Figure 4 shows the impedance profile changes for the Li/PEO_18_LiTFSI-*x*G4/Li cells at 60 °C as a function of storage time. PEO_18_LiTFSI-G4 and PEO_18_LiTFSI-1.5G4 show a significant increase of interface resistance with storage time; the initial cell resistance of 69 Ω·cm^2^ increased to 141 Ω·cm^2^ for PEO_18_LiTFSI-G4 after 28 days. However, low interface resistance was obtained for PEO_18_LiTFSI-2G4 and PEO_18_LiTFSI-3G4. After 28 days, PEO_18_LiTFSI-2G4 and PEO_18_LiTFSI-3G4 showed interface resistances of 34 and 74 Ω·cm^2^, respectively, which are much lower than 253 Ω·cm^2^ for PEO_18_LiTFSI after storage for 28 days [16]. The interface resistance behavior for PEO_18_LiTFSI-2G4 was unusual, where the interfacial resistance increased during the initial seven days and then decreased continuously to a value lower than the original. A similar change in the interface resistance was reported for the Li/PEGDME-LiTFSI/Li cell by Bernhard *et al*. [20], which could be attributed to the solid electrolyte interphase (SEI) formation-re-dissolution process [21]. These impedance profiles show a diminished semicircle, which is associated with the resistance of a passivation film (SEI) formed on the lithium electrode surface by the reaction of lithium with the polymer electrolyte and the charge transfer resistance. Figure 5 shows the temperature dependence of the inverse of the passivation film resistance and the charge transfer resistance for the Li/PEO_18_LiTFSI-*x*G4/Li cells as a function of *x*. The activation energy for the inverse of the passivation film resistance (*R*_p_) decreased from 76.2 kJ·mol^−1^ for PEO_18_LiTFSI to 54.7 kJ·mol^−1^ for PEO_18_LiTFSI-2G4. A decrease was also observed for PEO_18_LiTFSI-G4 and PEO_18_LiTFSI-3G4, but it was not as significant as that for PEO_18_LiTFSI-2G4. The activation energies for the inverse of the charge transfer resistance (*R*_c_) decreased with increasing *x*. The lowest value of 63.4 kJ·mol^−1^ was calculated for PEO_18_LiTFSI-3G4, in comparison with 81.7 kJ·mol^−1^ for PEO_18_LiTFSI and 68.1 kJ·mol^−1^ for PEO_18_LiTFSI-2G4. These results suggest that the low-molecular weight oligomer ether G4 could reduce the resistance of the SEI and facilitate the charge transfer reaction at the interface. The former role is similar to other additives, such as nanofillers and ionic liquids, whereas the latter role is only performed by G4. 

### 2.2. Electrochemical Performance of WSLEs

**Figure 6:**
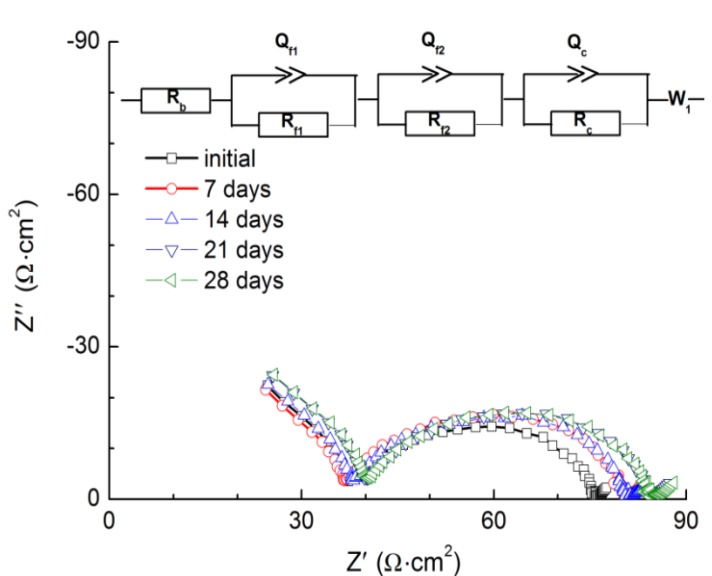
Impedance spectra of the Li/PEO_18_LiTFSI-2G4/LTAP/saturated LiCl aqueous solution/Pt, air cell as a function of the storage time at 60 °C.

The high lithium ionic diffusion coefficient and low interface resistance with lithium metal for PEO_18_LiTFSI-2G4 motivated us to examine its role as the protective layer in a WSLE. The impedance of the Li/PEO_18_LiTFSI-2G4/LTAP/sat. LiCl aqueous solution/Pt, air cell was measured at 60 °C, using a platinized platinum reference electrode. Figure 6 presents the impedance spectra of this cell for various storage times. The open circuit voltage (OCV) was stabilized at 3.48 V after one week, which is comparable with that reported previously (3.43 V) [8] and slightly lower than that of the calculated OCV (3.59 V). After 28 days, a stable cell resistance of 84 Ω·cm^2^ was obtained, which is comparable with those reported previously; the cell resistance for the Li/PEO_18_LiTFSI/LTAP/1 M LiCl/Pt, air cell was 539 Ω·cm^2^ [1], 118 Ω·cm^2^ for the Li/PEO_18_LiTFSI-40 nm BaTiO_3_/LTAP/1 M LiCl aqueous solution/Pt, air cell [22] and 130 Ω·cm^2^ for the Li/PEO_18_LiTFSI-1.44 *N*-methyl-*N*-propylpiperdinium-bis(trifluromethanesulfonyl)imide/LTAP/1 M LiCl aqueous solution/Pt, air cell [8]. An equivalent circuit proposed in our previous study was utilized to analyze the impedance spectra, which consists of the total resistances of the polymer electrolyte and the LTAP plate (*R*_b_), the interfacial resistance between the polymer electrolyte and lithium metal electrode (*R*_f1_), the interfacial resistance between the polymer electrolyte and the LTAP plate (*R*_f2_), the charge-transfer resistance (*R*_c_), and the Warburg impedance (*W*_1_) [14]. *R*_b_ was quite stable at around 38 Ω·cm^2^ during the storage period. The fitting results suggest that *R*_f2_ increased from 19.4 to 29.3 Ω·cm^2^, *R*_c_ decreased from 18.0 to 7.4 Ω·cm^2^, and *R*_f1_ increased from 3.8 to 5.8 Ω·cm^2^ over the 28-day storage period.

**Figure 7:**
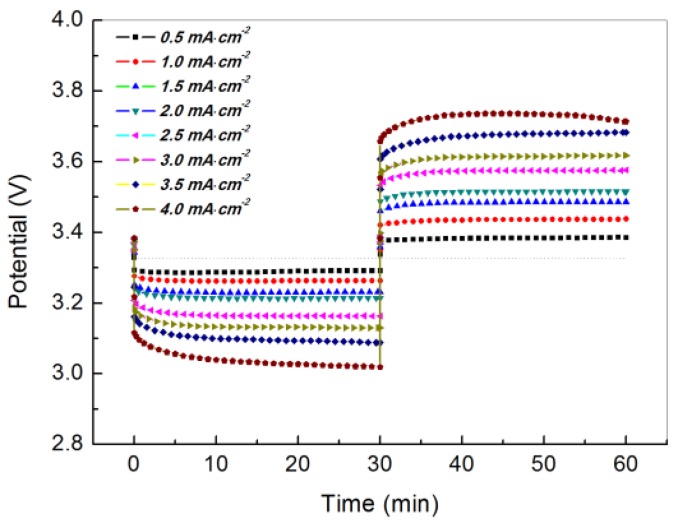
Discharge and charge profiles for the Li/PEO_18_LiTFSI-2G4/LTAP/1 M LiCl-4 mM LiOH aqueous solution/Pt, air cell at various current densities and 60 °C. The cell voltage was measured using a platinized platinum reference electrode.

The electrochemical performance of a WSLE protected by PEO_18_LiTFSI-2G4 and LTAP was investigated in an aqueous electrolyte using platinized platinum electrodes as the counter and reference electrodes. Figure 7 shows the change in potential over time for the Li/PEO_18_LiTFSI-2G4/LTAP/1 M LiCl-4 mM LiOH aqueous solution/Pt, air cell at current densities in the range of 0.5 to 4.0 mA·cm^−2^ at 60 °C. This WSLE exhibited quite low lithium plating and stripping overpotentials at high current densities up to 4.0 mA·cm^−2^. The lithium stripping and plating overpotentials at 1.5 mA·cm^−2^ were 0.10 and 0.15 V, respectively, which are lower than the best results obtained for the Li/PEO_18_LiTFSI-18 wt % PEGDME/LTAP/1 M LiCl-4 mM LiOH aqueous solution/Pt, air cell (0.29 V for plating and 0.21 V for stripping) in our previous studies [14]. The low overpotentials of the cell at high current densities could be attributed to the high lithium ion diffusion coefficient, high lithium ion transport number, and low interfacial resistance of PEO_18_LiTFSI-2G4.

**Figure 8:**
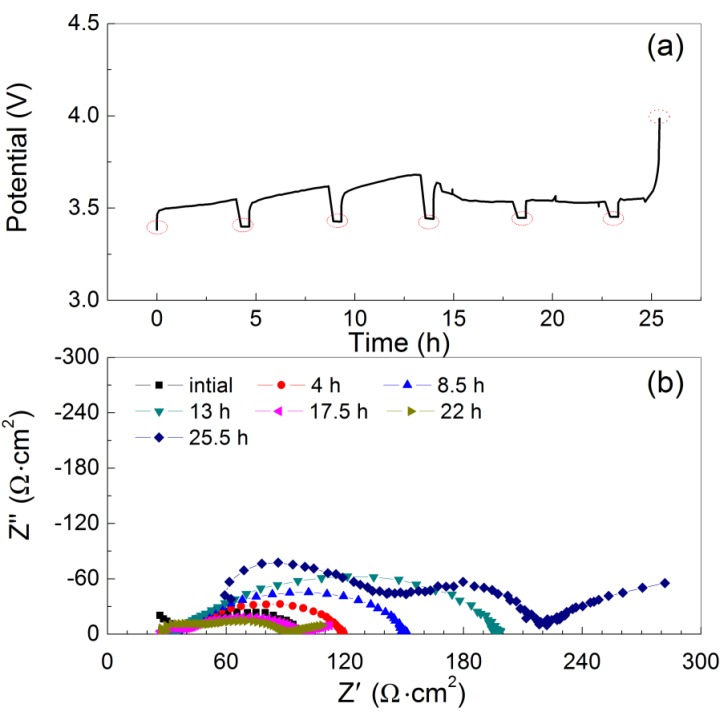
(**a**) Charge profiles for the Li/PEO_18_LiTFSI-2G4-100 μm/LTAP/sat. LiCl/Pt, air cell at 1 mA·cm^−2^ and 60 °C; and (**b**) impedance profiles after each polarization period. The cell voltage and impedance were measured using a platinized platinum air reference electrode.

To study the lithium dendrite formation at the interface of Li and PEO_18_LiTFSI-2G4, a long period of lithium deposition at a constant current of 1 mA·cm^−2^ was performed using the Li/PEO_18_LiTFSI-2G4/LTAP/saturated LiCl aqueous solution/Pt, air cell, where the thickness of PEO_18_LiTFSI-2G4 was around 100 μm and the impedance of the WSLE was measured at every 4 h polarization. Figure 8a shows cell potential *versus* time curves as a function of the polarization period. The lithium electrode potential increased suddenly after a 25 h polarization. This potential increase may be due to lithium deposition on LTAP by lithium dendrite formation, which would result in the formation of a high resistance layer by the reaction of lithium and LTAP [4]. Figure 8b shows the impedance profiles as a function of the polarization period. The WSLE resistance increased gradually with the polarization period up to 13 h and then decreased up to 22 h polarization. The decrease of electrode resistance may be due to lithium dendrite formation. Finally, the WSLE resistance was increased to 220 Ω·cm^2^ by the short circuit of the lithium metal electrode with LTAP. The short circuit period of 25 h is approximately 2.6 times longer than that measured for a Li/PEO_18_LiTFSI/LTAP/10 M LiCl-4 mM LiOH aqueous solution/Pt, air cell [14]. The long short circuit period for the Li/PEO_18_LiTFSI-2G4/LTAP/saturated LiCl aqueous solution/Pt, air cell could be explained by the high lithium ion transport number, high salt diffusion coefficient, and low interface resistance [17,23].

**Figure 9:**
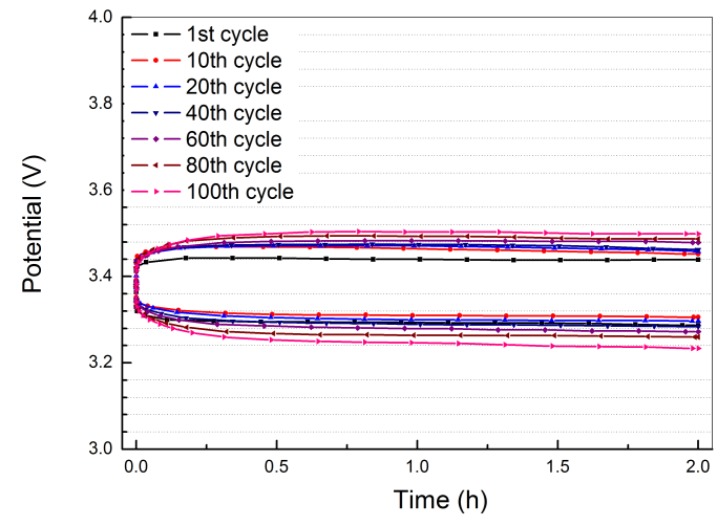
Discharge and charge profiles for Li/PEO_18_LiTFSI-2G4-100 μm/LTAP/sat. LiCl/Pt, air cell at 1.0 mA·cm^−2^ and 60 °C.

The cyclability of the Li/PEO_18_LiTFSI-2G4/LTAP/saturated LiCl aqueous solution/Pt, air cell for lithium deposition and stripping at a constant current density of 1.0 mA·cm^−2^ was measured at 60 °C, where the current was passed for 2 h. The cell voltage *versus* time profile is shown in Figure 9. After 100 cycles, the overpotential for lithium deposition and stripping slightly increased from 0.10 to 0.15 V and from 0.08 to 0.13 V, respectively. This excellent cycling performance could be ascribed to the low and stable resistance for the SEI formed between lithium metal and PEO_18_LiTFSI-2G4. 

## 3. Experimental Section

PEO (Sigma-Aldrich, M_v_ ≈ 600,000 g·mol^−1^) and LiTFSI (Wako Chemicals, Japan) were dried overnight under vacuum at 50 and 160 °C, respectively, and then transferred to an Ar-filled glove box without exposure to air. G4 (Kishida Chemical, M = 222.28 g·mol^−1^) was immersed into activated 0.3 nm molecular sieves for 1 week and the same pretreatment by molecular sieves was performed 3 times before G4 was stored in a glove box. The composite polymer electrolytes of PEO_18_LiTFSI-*x*TEGDME in the range of *x* = 0–3 were prepared using a casting method [24]. LiTFSI (Wako Chemicals, Japan) and G4 were added to an acetonitrile (AN) solution of PEO (Li/O = 1/18). The mixed solution was stirred at room temperature for 12 h and then cast into a clean Teflon dish. The AN solvent was evaporated slowly at room temperature in a Ar-filled dry glove box. After evaporation of the AN, the Teflon dish was transferred to a vacuum oven and dried at 80 °C for 24 h to remove residual AN. The resultant CPEs were *ca*. 100 μm thick.

Electrical conductivity measurements of the CPEs were performed using sandwich cells of Au/CPE/Au with gold foil electrodes. The cell impedances were measured using a frequency response analyzer (Solartron 1250) in the frequency range from 0.1 Hz to 1 MHz at temperatures in the range of 25–80 °C. Z-plot software was employed for data analysis.

The LTAP plate (*ca*. 250 μm thick) was supplied by Ohara Inc., Japan. The electrical conductivity of the LTAP plate at 60 °C was 1.4 × 10^−3^ S·cm^−1^. A Li/CPE/Li cell was assembled to investigate the interfacial resistance between lithium and the CPE. The active surface area was 1.00 cm^2^. The WSLE was prepared by sandwiching lithium metal foil (200 μm thick) with a Cu thin film lead, CPE, and the LTAP plate in a plastic envelope. The envelope was evacuated, heat-sealed apart from a 0.35 × 0.35 cm^2^ area, and then subjected to an isostatic pressure of 150 MPa for 15 min to ensure good contact. The WSLE was immersed into an aqueous solution of 1 M (or 10 M) LiCl with 4 mM LiOH. A platinized Pt electrode was used as both the counter and reference electrodes. Electrochemical measurements were conducted using a multichannel potentiostat-galvanostat (Biologic Science Instruments VMP3). The lithium ion transport number *t*_Li_^+^, was determined using the Li/CPE/Li cell with a combination of AC impedance spectroscopy and the DC polarization method, as originally proposed by Evans and Vincent, and later refined by Abraham and Jiang [19,25]. The cell potentials were measured after polarization for at least 4 h to obtain steady-state current data. The diffusion coefficient was estimated using a method proposed by Ma *et al*. [18]. The Li/CPE/Li cell was polarized at 50 mV under potentiostatic mode for a few hours to achieve steady-state, after which the potential was interrupted and monitored at 60 °C. 

## 4. Conclusions

The PEO_18_LiTFSI-2G4 electrolyte exhibited a high lithium ion conductivity of 8.91 × 10^−4^ S·cm^−1^, a high diffusion coefficient of 3.37 × 10^−7^ cm^2^·S^−1^ at 60 °C, and a low interfacial resistance in contact with lithium metal. The Li/PEO_18_LiTFSI-2G4/LTAP/saturated LiCl aqueous solution/Pt, air cell exhibited excellent cyclability. The overpotentials for lithium deposition and stripping at 2.0 mAh·cm^−2^ for 2 h polarization at 60 °C were increased slightly from 0.10 to 0.15 V and from 0.08 to 0.13 V after 100 cycles, respectively. Therefore, the CPE is an attractive material for the interlayer between lithium metal and LTAP for the water stable lithium electrode in lithium-air batteries.
